# The genus *Simplicillium*

**DOI:** 10.3897/mycokeys.60.38040

**Published:** 2019-11-19

**Authors:** De-Ping Wei, Dhanushka N. Wanasinghe, Kevin D. Hyde, Peter E. Mortimer, Jianchu Xu, Yuan-Pin Xiao, Chitrabhanu S. Bhunjun, Chaiwat To-anun

**Affiliations:** 1 Department of Entomology and Plant Pathology, Faculty of Agriculture, Chiang Mai University, Chiang Mai, 50200, Thailand; 2 Center of Excellence in Fungal Research, Mae Fah Luang University, Chiang Rai 57100, Thailand; 3 Key Laboratory for Plant Diversity and Biogeography of East Asia, Kunming Institute of Botany, Chinese Academy of Science, Kunming 650201, Yunnan, China; 4 Mushroom Research Foundation, 128 M.3 Ban Pa Deng T. Pa Pae, A. Mae Taeng, Chiang Mai 50150, Thailand; 5 World Agroforestry Centre, East and Central Asia, Kunming 650201, Yunnan, China; 6 Engineering Research Center of Southwest Bio-Pharmaceutical Resources, Ministry of Education, Guizhou University, Guiyang, Guizhou Province, 550025, China; 7 School of Science, Mae Fah Luang University, Chiang Rai, 57100, Thailand

**Keywords:** new species, Thailand, ant fungi, taxonomy, phylogeny

## Abstract

*Simplicillium* species have a wide host range and an extensive distribution. Some species are associated with rusts, as well as other plant pathogenic fungi and play an important role in biological control. In this study, two specimens of *Simplicillium* were collected from Chiang Mai Province, Thailand. *Simplicillium formicae* sp. nov. was isolated from an infected ant and *S. lanosoniveum* from *Ophiocordyceps unilateralis* which is a new host record. Species were initially identified using ITS gene sequences and confirmed using morphology coupled with phylogenetic analyses of a combined nrLSU, nrSSU, TEF and RPB1 dataset. *Simplicillium formicae* differs from other species in the genus by the presence of flask-shaped synnemata and phialides with intercalary nodes. *Simplicillium lanosoniveum* resembles other collections of the species by its completely solitary, tapering phialides and globose to ellipsoidal conidia which adhere in a slimly head. A key to species of *Simplicillium* is also provided.

## Introduction

Zare and Gams (2001) introduced *Simplicillium* to accommodate four taxa including the type species *S. lanosoniveum* and three other species, *S. lamellicola*, *S. obclavatum* and *S. wallacei*. *Simplicillium* species were historically placed in Verticillium sect. Prostrata which was described by Gams (1971) for prostrate conidiophore-producing species. Later, most of the species of Verticillium sect. Prostrata were reported as members in Clavicipitaceae, based on molecular data (including SSU, LSU and ITS sequences), whereas *Simplicillium* species consistently formed a monophyletic group apart from the other described taxa in this family (Zare et al. 2000; Gams and Zare 2001; Sung et al. 2001; Zare and Gams 2001). Recently, Clavicipitaceae was divided into three families, based on multi-gene phylogenetic analyses and *Simplicillium* was assigned to Cordycipitaceae (Hypocreales, Hypocreomycetidae, Sordariomycetes) (Sung et al. 2007; Maharachchikumbura et al. 2016; Wijayawardene et al. 2018). Zare and Gams (2008) excluded *Simplicillium wallacei* from *Simplicillium* and transferred it to *Lecanicillium* due to the basal position being closer to the latter genus than to the former genus in the cladogram of ITS data. Subsequently, ten species viz. *Simplicillium chinense* (Liu and Cai 2012), *S. aogashimaense*, *S. cylindrosporum*, *S. minatense*, *S. subtropicum*, *S. sympodiophorum* (Nonaka et al. 2013), S. lanosoniveum var. tianjinensis (Dong et al. 2014), *S. calcicola* (Zhang et al. 2017), *S. coffeanum* (Gomes et al. 2018) and *S. filiforme* (Crous et al. 2018) were restricted to *Simplicillium*, based on the phylogenetic analyses of ITS sequence data and strong morphological evidence. Its sexual-asexual connection has been established with *S. lanosoniveum* linked to a *Torrubiella* sp. (Zare and Gams 2001).

*Simplicillium* species have a wide distribution and are considered as mammal and plant-parasitic, symbiotic, entomopathogenic, fungicolous and nematophagous fungi, as they have a broad spectrum of hosts and substrates, such as insects, plants, rusts, nematodes, human nails, canine tissues and mushrooms, *Chroococcus* sp., soil, freshwater, marine and terrene environments (Zare and Gams 2001; Guo et al. 2012; Liu and Cai 2012; Dong et al. 2014; Liang et al. 2016; Sun et al. 2019). Several studies have been shown that *Simplicillium* species have a high ecological and economical value for biocontrol and bioactive compounds (Takata et al. 2013; Yan et al. 2015; Hyde et al. 2019). For example, *Simplicillium lanosoniveum* can be a phytopathogen, causing brown spots and lesions on flowers (Chen et al. 2008) or a mycoparasite on soybean rust (Ward et al. 2012; Gauthier et al. 2014) or a pathogen on aphids and other phytopathogens (Chen et al. 2017) or an anti-*Trichomonas vaginalis* agent (Scopel et al. 2013). *Simplicillium chinense* can be a biological control agent against plant parasitic nematodes (Zhao et al. 2013; Luyen 2017). *Simplicillium lamellicola* can suppress plant bacterial diseases and grey mould diseases of tomato (*Solanum lycopersicum*) and ginseng (*Panax ginseng*) (Dang et al. 2014; Shin et al. 2017). *Simplicillium obclavatum* has the ability to produce multiple xylanases and endoglucanases that have the potential to be used in biofuels, animal feed and food industry applications (Roy et al. 2013). Bioactive compounds with anti-fungal and anti-bacterial profiles and pharmaceutical exopolysaccharides have been isolated from *S. lanosoniveum* (Yu et al. 2013; Fukuda et al. 2014; Xing et al. 2016; Dong et al. 2018). Linear and cyclic peptides with anti-fungal and anti-viral properties have also been discovered from the secondary metabolites of *S. obclavatum* (Liang et al. 2016, 2017).

Recent studies have shown that Thailand supports an amazing fungal diversity with numerous new species that have the potential for biotechnological application (Hyde et al. 2018, 2019). In this study, we introduce a novel species, *Simplicillium formicae* from northern Thailand and a new record of *S. lanosoniveum* with evidence from a combination of molecular analyses and morphological characteristics to reserve a natural resource for future studies regarding biocontrol in the forestry, agricultural and pharmaceutical industries.

## Material and methods

### Sample collection and isolation

The Mushroom Research Centre (MRC) is a disturbed rainforest located in Chiang Mai Province, Thailand (Aung et al. 2008). The forest consists of various tall tree and lower shrubs. The climate of Chiang Mai is controlled by tropical monsoons and the weather is typically hot and humid with temperatures often close to or above 30 °C. Frequent rain and thunder showers usually last from June to late October (Chiang Mai Buddy website: https://chiangmaibuddy.com/welcome-to-chiang-mai/weather-and-climate/, accessed 26.8.2019). Two ant fungi were found anchored to the underside of two different shrubby leaves in the forest at the Mushroom Research Centre. These two fresh specimens; HKAS 102459 and HKAS 102447 were collected and placed in plastic containers and transported to the laboratory for subsequent study. Interestingly, the ant fungus HKAS 102447 was already dead and was colonised by a saprobic fungus. The isolate MFLUCC 18–1385 was separated from this saprobe which occurred on the surface of specimen HKAS 102447 via single spore isolation. The isolate MFLUCC 18–1379 was separated from specimen HKAS 102459 by directly cultivating the hyphae which covered the surface of the ant host. These two isolates were cultured with potato dextrose agar (PDA, 1% w/v peptone) and incubated at room temperature (25 °C).

### Morphological studies

For long-term deposit, these two specimens were dried with allochroic silica gel to protect them from contamination of opportunistic fungi and to retain the informative taxonomic characters. The macro-morphological characters were observed with a stereoscope (Olympus SZ61) and the micro-morphological features were examined with a compound microscope (Nikon ECLIPSE Ni). Important characteristics such as mycelium, phialides and conidia were captured with a digital camera (Canon EOS 600D). Measurements of perithecia, synnemata, phialides and conidia were taken using the Tarosoft (R) Image Frame Work programme and the images used were processed with Adobe Photoshop CS3 Extended v. 10.0 (Adobe, San Jose, CA).

### DNA extraction, PCR amplification and sequencing

DNA was extracted from fresh mycelium of isolates MFLUCC 18–1379 and MFLUCC 18–1385 and from stromal tissue of ant fungus HKAS 102447 (the host of isolate MFLUCC 18–1385) using a DNA extraction kit (Biospin Fungus Genomic DNA Extraction Kit, BioFlux, China), following the instructions of the manufacturer. Extracted DNA was stored at 4 °C for use in regular work and duplicated at –20 °C for long-term storage. The internal transcribed spacer (ITS1-5.8S-ITS2, ITS) was amplified with primer ITS4 and ITS5 (White et al. 1990) and was used for individual gene phylogenetic analyses. The large subunit (LSU), small subunit rDNA (SSU), translation elongation factor 1-alpha gene (TEF1-α) and RNA polymerase II largest subunit 1 (RPB1) were also amplified as described in Wei et al. (2018) and used for multi-gene phylogenetic analyses. The PCR products were sent to Sangon Company, Kunming City, Yunnan Province, China for sequencing using the above primers. Newly generated sequences, used in the study, were submitted to GenBank to be assigned their accession numbers.

### Sequence alignments and phylogenetic analyses

The raw sequences were verified with Finch TV version 1.4.0 (Mccredden 2016) and assembled with BioEdit v. 7.0.9.1 (Hall 1999). Sequence data were downloaded from GenBank based on BLAST searches of ITS sequences and with reference to the recent publications (Table 1). Most *Simplicillium* species are lacking protein-coding genes, but ITS sequences are available for all the species that are useful in understanding the intraspecific relationships within *Simplicillium* (Liu and Cai 2012, Nonaka et al. 2013, Dong et al. 2014 and Crous et al. 2018). Therefore, phylogenetic analyses, based on ITS regions, were generated throughout *Simplicillium* for the primary identification. Multi-gene phylogenetic analysis of the combined SSU, LSU, TEF and RPB1 sequences from representative species in Hypocreales was afterwards performed to confirm the taxonomic placements of our isolates.

**Table 1.** Strains and GenBank accession numbers from related references used in multi-gene tree.

Taxon	Voucher no.	Host/substrate	SSU rRNA	LSU rRNA	tef1	rpb1	Reference
*Akanthomyces tuberculata*	OSC 111002	Lepidoptera	DQ522553	DQ518767	DQ522338	DQ522384	Spatafora et al. (2007)
*Aschersonia badia*	BCC 8105	Scale insect	DQ522537	DQ518752	DQ522317	DQ522363	Spatafora et al. (2007)
*Aschersonia placenta*	BCC 7957	Scale insect	DQ522538	DQ518753	DQ522318	DQ522364	Spatafora et al. (2007)
*Balansia henningsiana*	GAM 16112=AEG96-27a	*Panicum* sp.	AY545723	AY545727	AY489610	AY489643	Spatafora et al. (2007)
*Balansia pilulaeformis*	AEG 94-2	Poaceae	AF543764	AF543788	DQ522319	DQ522365	Spatafora et al. (2007)
*Claviceps fusiformis*	ATCC 26019	Poaceae	DQ522539	U17402	DQ522320	DQ522366	Spatafora et al. (2007)
*Claviceps paspali*	ATCC 13892	Poaceae	U32401	U47826	DQ522321	DQ522367	Spatafora et al. (2007)
*Claviceps purpurea*	GAM 12885	*Dactylis glomerata*	AF543765	AF543789	AF543778	AY489648	Spatafora et al. (2007)
*Cordyceps farinosa*	OSC 111005	Lepidoptera pupa	DQ522558	DQ518773	DQ522348	DQ522394	Spatafora et al. (2007)
*Cordyceps heteropoda*	OSC 106404	Cicada	AY489690	AY489722	AY489617	AY489651	Spatafora et al. (2007)
*Cordyceps militaris*	OSC 93623	Lepidoptera	AY184977	AY184966	DQ522332	DQ522377	Spatafora et al. (2007)
*Cordyceps ophioglossoides*	OSC 106405	*Elaphomyces* sp.	AY489691	AY489723	AY489618	AY489652	Spatafora et al. (2007)
*Cordyceps pruinosa*	ARSEF 5413	*Iragoides fasciata*	AY184979	AY184968	DQ522351	DQ522397	Spatafora et al. (2007)
*Cordyceps scarabaeicola*	ARSEF 5689	Scarabaeidae pupa	AF339574	AF339524	DQ522335	DQ522380	Spatafora et al. (2007)
*Cordyceps tenuipes*	OSC 111007	Lepidoptera pupa	DQ522559	DQ518774	DQ522349	DQ522395	Spatafora et al. (2007)
*Drechmeria balanoides*	CBS 250.82	Nematoda	AF339588	AF339539	DQ522342	DQ522388	Spatafora et al. (2007)
*Drechmeria gunnii*	OSC 76404	Lepidoptera larva	AF339572	AF339522	AY489616	AY489650	Spatafora et al. (2007)
*Drechmeria sinensis*	CBS 567.95	Nematoda	AF339594	AF339545	DQ522343	DQ522389	Spatafora et al. (2007)
*Engyodontium aranearum*	CBS 309.85	Spider	AF339576	AF339526	DQ522341	DQ522387	Spatafora et al. (2007)
*Epichloë typhina*	ATCC 56429	*Festuca rubra*	U32405	U17396	AF543777	AY489653	Spatafora et al. (2007)
*Hypocrella nectrioides*	GJS 89-104	Scale insect	U32409	U47832	DQ522347	DQ522393	Spatafora et al. (2007)
*Hypocrella schizostachyi*	BCC 14123	Scale insect	DQ522557	DQ518771	DQ522346	DQ522392	Spatafora et al. (2007)
*Lecanicillium antillanum*	CBS 350.85	Agaric	AF339585	AF339536	DQ522350	DQ522396	Spatafora et al. (2007)
*Lecanicillium lecanii*	CBS 101247=IMI 304807	*Coccus viridis*	AF339604	AF339555	DQ522359	DQ522407	Spatafora et al. (2007)
*Lecanicillium wallacei*	CBS 101237=IMI 331549	Lepidoptera		AY184967	EF469073	EF469102	Zare and Gams (2008); Kouvelis et al. (2008)
*Metacordyceps chlamydosporia*	CBS 101244	Mollusca	DQ522544	DQ518758	DQ522327	DQ522372	Spatafora et al. (2007)
*Metacordyceps taii*	ARSEF 5714	Lepidoptera	AF543763	AF543787	AF543775	DQ522383	Spatafora et al. (2007)
*Metapochonia goniodes*	CBS 891.72	Nematoda	AF339599	AF339550	DQ522354	DQ522401	Spatafora et al. (2007)
*Metarhizium album*	ARSEF 2082	*Cofana spectra*	DQ522560	DQ518775	DQ522352	DQ522398	Spatafora et al. (2007)
*Metarhizium flavoviride*	ARSEF 2037	*Nilaparvata lugens*	AF339580	AF339531	DQ522353	DQ522400	Spatafora et al. (2007)
*Metarhizium majus*	ARSEF 3145	*Oryctes rhinoceros*	AF339579	AF339530	AF543774	DQ522399	Spatafora et al. (2007)
*Myriogenospora atramentosa*	AEG 96-32	*Andropogon virginicus*	AY489701	AY489733	AY489628	AY489665	Spatafora et al. (2007)
*Ophiocordyceps acicularis*	OSC 128580	Coleoptera	DQ522543	DQ518757	DQ522326	DQ522371	Araújo et al. (2018)
*Ophiocordyceps aphodii*	ARSEF 5498	Coleoptera	DQ522541	DQ518755	DQ522323		Araújo et al. (2018)
*Ophiocordyceps brunneipunctata*	OSC 128576	Coleoptera	DQ522542	DQ518756	DQ522324	DQ522369	Araújo et al. (2018)
*Ophiocordyceps irangiensis*	OSC 128577	Ant	DQ522546	DQ518760	DQ522329	DQ522374	Araújo et al. (2018)
*Ophiocordyceps irangiensis*	OSC 128578	Ant	DQ522556	DQ518770	DQ522345	DQ522391	Araújo et al. (2018)
*Ophiocordyceps melolonthae*	OSC 110993	Scarabaeidae larva	DQ522548	DQ518762	DQ522331	DQ522376	Araújo et al. (2018)
*Ophiocordyceps nutans*	OSC 110994	Stink bug	DQ522549	DQ518763	DQ522333	DQ522378	Araújo et al. (2018)
*Ophiocordyceps ravenelii*	OSC 110995	*Phyllophaga* sp.	DQ522550	DQ518764	DQ522334	DQ522379	Araújo et al. (2018)
*Ophiocordyceps sphecocephala*	OSC 110998	Wasp	DQ522551	DQ518765	DQ522336	DQ522381	Araújo et al. (2018)
*Ophiocordyceps stylophora*	OSC 111000	Elateridae grub	DQ522552	DQ518766	DQ522337	DQ522382	Araújo et al. (2018)
*Ophiocordyceps unilateralis*	OSC 128574	Ant	DQ522554	DQ518768	DQ522339	DQ522385	Araújo et al. (2018)
*Ophiocordyceps variabilis*	ARSEF 5365	Diptera larva	DQ522555	DQ518769	DQ522340	DQ522386	Spatafora et al. (2007)
*Rotiferophthora angustispora*	CBS 101437	Rotifera	AF339584	AF339535	AF543776	DQ522402	Spatafora et al. (2007)
*Simplicillium calcicola*	LC5586 = CGMCC3.17943	Calcaire	KY883301	KU746752	KX855252		Spatafora et al. (2007)
*Simplicillium lamellicola*	CBS 116.25	*Agaricus bisporus*	AF339601	AF339552	DQ522356	DQ522404	Spatafora et al. (2007)
*Simplicillium lanosoniveum*	CBS 704.86	*Hemileia vastatrix*	AF339602	AF339553	DQ522358	DQ522406	Spatafora et al. (2007)
*Simplicillium obclavatum*	CBS 311.74	Air above sugarcane filed	AF339567	AF339517	EF468798		Spatafora et al. (2007)
*Tolypocladium fractum*	OSC 110990	*Elaphomyces* sp.	DQ522545	DQ518759	DQ522328	DQ522373	Spatafora et al. (2007)
*Tolypocladium japonicum*	OSC 110991	*Elaphomyces* sp.	DQ522547	DQ518761	DQ522330	DQ522375	Spatafora et al. (2007)
*Torrubiella ratticaudata*	ARSEF 1915	*Euophrys* sp.	DQ522562	DQ518777	DQ522360	DQ522408	Spatafora et al. (2007)
*Verticillium epiphytum*	CBS 384.81	*Hemileia vastatrix*	AF339596	AF339547	DQ522361	DQ522409	Spatafora et al. (2007)

The generated sequences of each gene region were aligned separately with representative sequences using MAFFT v. 7 web server (http://mafft.cbrc.jp/alignment/server) (Kuraku et al. 2013; Katoh et al. 2017). The uninformative gaps and ambiguous regions were manually removed and different gene regions were concatenated using BioEdit v. 7.0.9.1 (Hall 1999). The maximum Likelihood (ML) analyses was performed using RAxML-HPC2 on XSEDE (8.2.10) at CIPRES Science Gateway V. 3.3 (https://www.phylo.org/portal2/home.action), with default setting, except the bootstrap iterations were set to 1,000 and the substitution model set to GTRGAMMA + I (Miller and Blair 2009). Maximum Parsimony analysis (MP) was performed by PAUP v. 4.0b10 (Swofford 2002) with the heuristic search option and Tree-Bisection-Reconnection (TBR) branch-swapping algorithm for 1000 random replicates. Branches that have a minimum branch length of zero were collapsed. Gaps were treated as “missing” and starting tree(s) were generated via stepwise addition (Hillis and Bull 1993). Tree Length [TL], Consistency Index [CI], Retention Index [RI], Rescaled Consistency Index [RC] and Homoplasy Index [HI]) were calculated for all parsimonious trees. For Bayesian analysis, the best models of each gene were selected under Akaike Information Criterion (AIC) employing MrModeltest v. 2.3 (Nylander et al. 2008) and PAUP v. 4.0b10 (Ronquist and Huelsenbeck 2003). Bayesian analysis was performed using MrBayes v. 3.1.2 (Rannala and Yang 1996; Zhaxybayeva and Gogarten 2002) to evaluate posterior probabilities (BYPP) with the Markov Chain Monte Carlo sampling (MCMC) method. Trees were sampled and printed to output at every 1000 generations. The first 25% of sampled trees were discarded as part of a burn-in procedure, the rest of the trees were used to create the consensus tree and the average standard deviation of split frequencies was set as 0.01.

Phylogenetic trees were visualised with FigTree v1.4.0 (Rambaut 2006) and edited in Microsoft PowerPoint, then saved as a PDF format and finally altered to JPG format using Adobe Illustrator CS6 (Adobe Systems Inc., United States). The finalised alignments and trees were submitted in TreeBASE (http://www.treebase.org/), with submission ID 24238 (ITS) and 24240 (multi-gene).

## Results and discussion

### Phylogenic analysis

**Figure 1.**
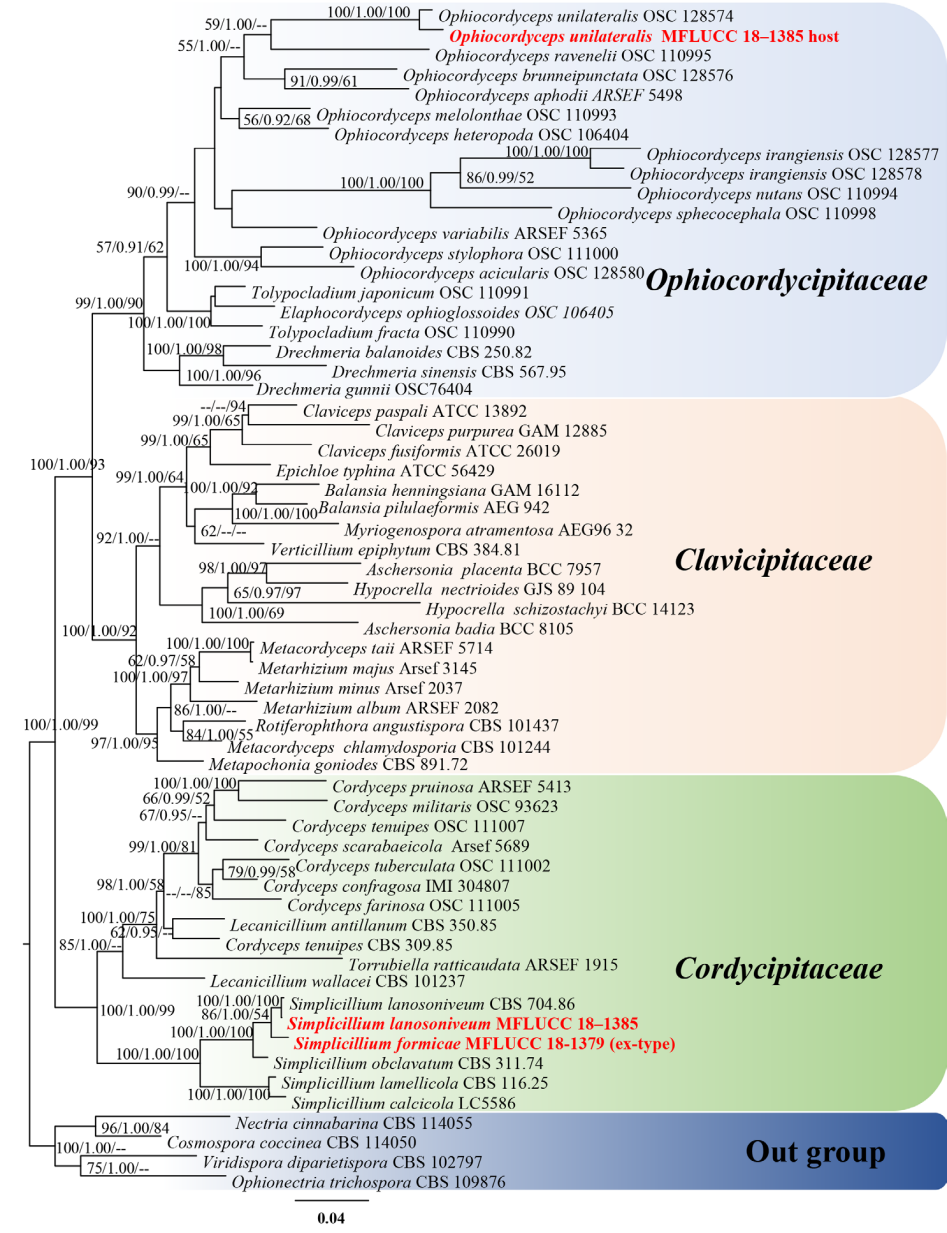
Phylogram generated from maximum likelihood analysis based on combined SSU, LSU, TEF and RPB1 sequence data. Bootstrap values for maximum likelihood (ML, left) and maximum parsimony (MP, right) equal to or greater than 50% and Bayesian posterior probabilities (BYPP, middle) equal to or greater than 0.90 are placed nearby the note. The newly generated sequences are indicated in red bold.

The combined four gene dataset comprised 60 taxa from three families (Cordycipitaceae, Ophiocordycipitaceae and Clavicipitaceae) (Table 1) with *Cosmospora coccinea*, *Nectria cinnabarina*, *Ophionectria trichospora* and *Viridispora diparietispor*a as the outgroup taxa. The RAxML analysis of the combined dataset yielded a best scoring tree (Figure 1) with a final ML optimisation likelihood value of −39792.595439. The alignment comprised 3469 total characters including gaps, of which 2077 were constant, 338 variable characters parsimony-uninformative and 1054 characters parsimony-informative. The Kishino-Hasegawa (KH) test showed CI = 0.281, RI = 0.527, RC = 0.148 and HI = 0.719. The matrix had 1655 distinct alignment patterns, with 6.42% undetermined characters or gaps. Estimated base frequencies were as follows: A = 0.241091, C = 0.260362, G = 0.272837, T = 0.225710; substitution rates AC = 0.985172, AG = 2.843760, AT = 0.887714, CG = 0.898140, CT = 6.284116, GT = 1.000000; gamma distribution shape parameter α = 0.585080. MrModeltest v. 2.3 imply that GTR+I+G is the best-fit model for LSU and RPB1, SYM+I+G for SSU and TEF sequences.

**Figure 2.**
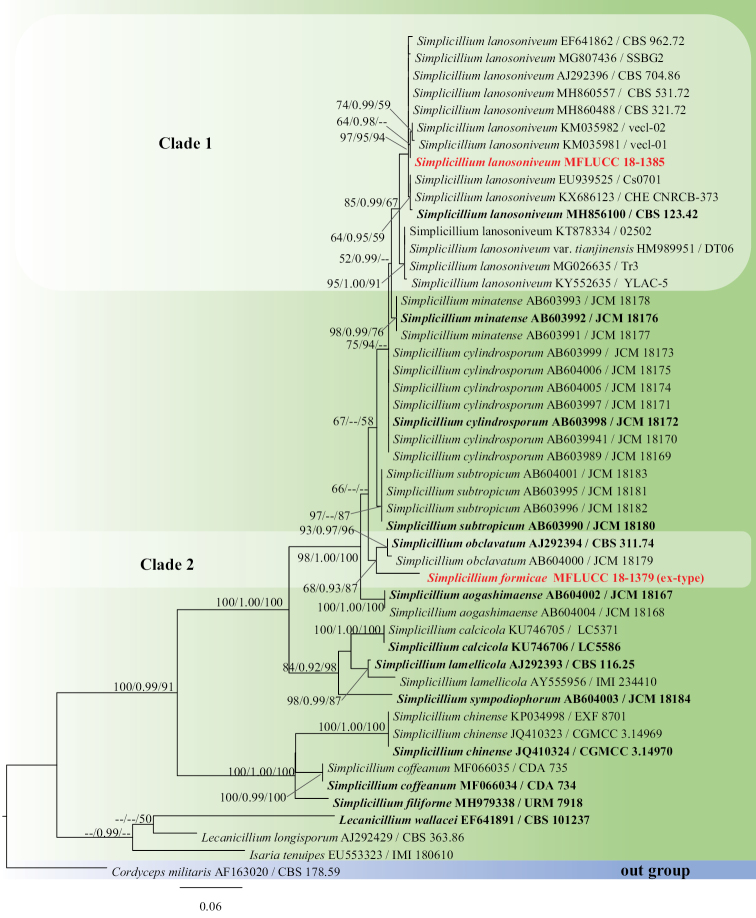
Phylogram generated from maximum likelihood analysis based on ITS sequence data. Bootstrap values for maximum likelihood (ML, left) and maximum parsimony (MP, right) equal to or greater than 50% and Bayesian posterior probabilities (BYPP, middle) equal to or greater than 0.90 are placed nearby the branches, respectively. The newly generated sequences are indicated in red bold and the type species are highlighted in black bold.

The ITS dataset comprised 49 taxa from all *Simplicillium* species that are currently available in GenBank (Figure 2) with *Cordyceps militaris* (CBS178.59) (Cordycipitaceae, Hypocreales) as the outgroup taxon. The RAxML analysis of the ITS dataset yielded a best scoring tree (Figure 2) with a final ML optimisation likelihood value of −3155.597177. The alignment comprised 570 total characters including gaps, of which 346 were constant, 43 variable characters parsimony-uninformative and 181 characters parsimony-informative. The Kishino-Hasegawa (KH) test showed CI = 0.681, RI = 0.856, RC = 0.583 and HI = 0.319. The matrix had 283 distinct alignment patterns, with 6.45% undetermined characters or gaps. Estimated base frequencies were as follows: A = 0.232003, C = 0.283823, G = 0.254774, T = 0.229400; substitution rates AC = 2.623562, AG = 2.645665, AT = 2.248749, CG = 1.653083, CT = 5.842034, GT = 1.000000; gamma distribution shape parameter α = 0.980038. MrModeltest v. 2.3 imply that GTR+I+G is the best-fit model for ITS sequences.

The multi-gene phylogenetic analyses showed that our isolates MFLUCC 18-1379 and MFLUCC 18–1385 grouped with the remaining *Simplicillium* species with strong support (100% ML, 1.00 BYPP, 100% MP, Figure 1) in Cordycipitaceae. The host of isolate MFLUCC 18–1385 grouped with *Ophiocordyceps unilateralis* (OSC 128574) in Ophiocordycipitaceae with a significant statistical support (100% ML, 1.00 BYPP, 100% MP, Figure 1). In the individual ITS-based phylogenetic tree, the isolate MFLUCC 18-1379 constituted a close affiliation to *Simplicillium obclavatum* with moderate bootstrap support (68% ML, 0.93 BYPP, 87% MP, Figure 2, clade 2). The fungal isolate MFLUCC 18-1385 grouped with the remaining *Simplicillium lanosoniveum* strains with 85% ML, 0.99 BYPP and 67% MP support (Figure 2, clade 1).

### Taxonomy

#### *Simplicillium* W. Gams & Zare, Nova Hedwigia 73(1-2): 38 (2001)

Hyperparasitic on rusts or parasitic on nematodes or occurring in soil. ***Asexual morph***: *Mycelium* thin, hyaline, septate, branched, smooth-walled. *Phialides* arising from prostrate aerial hyphae or rope-like and flask-shaped synnemata, typically solitary, rarely in whorls of 2–3, gradually tapering towards the apex, elongate, slender, smooth-walled, phialidic. *Conidia* hyaline, oval, spindle-shaped, cylindrical, subglobose to ellipsoidal, fusoid to filiform, straight to curved, smooth-walled. Conidia commonly form in small globose heads, sometimes in branched, unbranched, zigzag or imbricate chains, occasionally in sympodial proliferation with cylindrical conidium-bearing denticles. Colonies of species in this genus are usually fast growing, reaching 10–38 mm within 10 days on PDA, white, reverse brownish-cream to pale yellow, margin entire, cottony, fluffy or floccose. Some species produce yellow or orange pigment. Crystals are commonly present in the agar. ***Sexual morph***: *Torrubiella* (Zare and Gams 2001; Liu and Cai 2012; Nonaka et al. 2013; Dong et al. 2014; Gomes et al. 2018; Zhang et al. 2017).

In this study, we introduce a new species and a new host species as described below.

##### Simplicillium formicae

Taxon classificationFungiHypocrealesCordycipitaceae

D.P. Wei & K.D. Hyde sp. nov.

DC1DBEE1-68F2-53E1-A4BA-5AF04F7CCBDE

Figure 3 , 4

###### Etymology.

the epithet refers to its host–ant.

###### Holotype.

HKAS 102459; living culture: MFLUCC 18–1379.

###### Description.

Parasitic on ant (Formicidae). ***Asexual morph***: Hyphomycetous. *Mycelium* rarely septate, hyaline, smooth-walled, covering the whole body of the ant host. *Synnemata* 250–350 × 65–100 (xˉ = 300 × 86, n = 10) µm, forming at the head region of ant host in circular arrangement, flask-shaped, hyaline to yellowish, composed of dense hyphae, somehow curved. *Phialides* 25–100 × 0.5–1.5 (xˉ = 49 × 1.1, n = 20) µm, arising from procumbent hyphae or synnemata, blastic, enteroblastic, phialidic, monophialidic, discrete, terminal, unbranched, solitary, aseptate, hyaline, smooth-walled, slender, occasionally a swollen node present. *Conidia* 2–3.5 × 1.5–2.5 (xˉ = 2.6 × 2, n = 30) µm, globose to ellipsoidal, hyaline, one-celled, smooth-walled, round at both ends, adhering in slimy head on the tip of phialides. ***Sexual morph***: Undetermined.

###### Culture characteristics.

The colonies were rapid-growing on PDA medium, reaching a diameter of 2.5–3 (xˉ = 2.6, n = 9) cm, in 13 days at 22 °C, entire margin, circular, velvety and white from above, with radial crack and primrose-yellow on reverse. *In vitro*, *Synnemata* absent. *Phialides* 25–75 × 0.4–0.6 (xˉ = 50 × 0.55, n = 10) µm, arising from procumbent hyphae, blastic, enteroblastic, phialidic, discrete, terminal, unbranched, solitary, aseptate, hyaline, smooth-walled, relatively slender and long. *Conidia* 1.5–3 × 1.5–2.5 (xˉ = 2.3 × 1.7, n = 100) µm, hyaline, globose to ellipsoidal, aseptate, smooth-walled, slightly guttulate, adhering in slimy head on the tip of phialides.

###### Material examined.

THAILAND, Chiang Mai Province, Mushroom Research Centre, on an adult ant, 1 April 2018, *Deping Wei*, MRC18040102 (***holotype***: HKAS 102459; ***ex-type living culture***: MFLUCC 18–1379). Sequences generated from this strain have been deposited in GenBank with accession numbers: SSU = MK765046, LSU = MK766512, ITS = MK766511, TEF = MK926451, RPB1 = MK882623.

###### Note.

Isolate MFLUCC 18–1379 has a close phylogenetic relationship with *Simplicillium obclavatum*, based on ITS sequence analysis. The new isolate is similar to *Simplicillium obclavatum* in terms of shape and dimensions of the conidia with slender phialides tapering towards the apex. However, they have a different conidial arrangement, by *Simplicillium obclavatum* having short-imbricate chains, whereas the new fungus has subglobose to globose head. There are numerous synnemata in a circular arrangement which can be observed in our isolate and those are absent in *Simplicillium obclavatum*. The comparisons of ITS sequences between our isolate MFLUCC 18–1379 and ex-type strain of *Simplicillium obclavatum* (CBS 311.74) show 23 bp differences within 550 bp (4.2%). Thereby, we identify our isolates as a new species according to Jeewon and Hyde (2016).

##### Simplicillium lanosoniveum

Taxon classificationFungiHypocrealesCordycipitaceae

(J.F.H. Beyma) Zare & W. Gams, Nova Hedwigia 73(1–2): 39 (2001)

19637BCC-2B6E-518D-B473-15F078A0C21F

Facesoffungi number: FoF 05814

Index Fungorum number: 532459 Figure 5

Cephalosporium lanosoniveum J.F.H. Beyma, Antonie van Leeuwenhoek 8: 121 (1942) (Basionym)

###### Ex-type.

Netherlands, on hair of *Cibotium schiedei* in greenhouse, 1942, F.H. van Beyma, CBS123.42.

###### Description.

Saprophytic on *Ophiocordyceps unilateralis*. ***Asexual morph***: Hyphomycetous. *Mycelium* aseptate, hyaline, smooth-walled. *Phialides* 20–40 × 1.1–2 (xˉ = 30 × 1.6, n = 20) µm, arising from the prostrate mycelium, blastic, enteroblastic, phialidic, monophialidic, discrete, terminal, aseptate, hyaline, smooth-walled, solitary, tapering toward the apex. *Conidia* 2–4.5 × 1–3 (xˉ = 3 × 1.8, n = 60) µm, hyaline, amerospores, globose to ellipsoidal, smooth-walled, adhering in globose to ellipsoidal head at the apex of phialides. ***Sexual morph***: Undetermined.

###### Culture characters.

The colonies on PDA medium were rapid-growing, reaching a diam. of 5.5 cm in 30 days at 22 °C, white, entire margin, velvety, with radial cracks and primrose-yellow on the reverse.

Host and distribution: Saprophytic on fungi, endophytic or symbiotic or pathogenic on plant, parasitic on rust, nematode and insect, occurring on soil, animal hair or human bronchoalveolar lavage fluid, with a cosmopolitan distribution (see Table 2).

**Table 2.** Distribution, host and available sequence data of *Simplicillium lanosoniveum* strains.

Species	Strain no.	Host and habitat	Origin	Available gene region	Morphological description	Reference
*S. lanosoniveum*	CBS123.42	Hair of *Cibotium schiedei* (Plant)	Netherland	ITS, LSU		GenBank; Zare and Gams (2001)
	Cs0701	*Salvinia molesta* (Plant)	Taiwan	ITS	√	Chen et al. (2008)
	**PSU-ES104**	***Enhalus acoroides* (Plant)**	**Trang Province, Thailand**	**ITS**		**Supaphon et al. (2014)**
	CBS 531.72	*Salvinia rotundifolia* (Plant)	USA	ITS		Zare and Gams (2001)
	Tr3	*Salvia miltiorrhiza* (Plant)	China	ITS		GenBank
	YLAC-5	Endophytic on *Inula aconitum* (Plant)	China	ITS		GenBank
		Endophytic on seaweed (Plant)	India	SSU		GenBank
	E1, E3, E5	Endophytes of *Sophora alopecuroides* (Plant)	Ningxia, China	SSU, ITS	√	Yu et al. (2013)
	GA-B1	*Grewia asiatica* (Plant)	Shivalik region, Jammu, India	SSU		GenBank
	IMI 303103b	*Hemileia vastatrix* (Rust)	Colombia	ITS, SSU		Zare and Gams (2001), Kouvelis et al. (2008)
	AMH 9654	Rust pustules on leaves of *Elaeagnus* sp.	India	LSU, ITS	√	Baiswar et al. (2014)
	D082307-2A	Soybean rust	Louisiana	ITS	√	Ward et al. (2011)
	vecl-02	Rust of *Eleagnus latifolia*	India	ITS		GenBank
	vecl-01	Rust of *Eleagnus latifolia*	India	ITS		GenBank
	CBS 704.86	*Hemileia vastatrix* (Rust)	Venezuela	ITS, SSU, LSU, TEF, RPB1, RPB2, ATP	√	GenBank; Zare et al. (2000)
	S-599	*Coleosporium plumeriae* (Rust)	Campos dos Goytacazes, GJ, Brazil	ITS		Berlanga-Padilla et al. (2018)
	D082307-2A-GFP15	*Phakopsora pachyrhizi* (Rust)	Florida, USA		√	Gauthier et al. (2014)
	**HKAS 102447**	***Ophiocordyceps unilateralis* (Fungi)**	**Chiang Mai, Thailand**	**SSU, LSU, ITS, TEF, RPB1**	√	**This study**
	TYL001	*Pseudaulacaspis pentagona* (Insect)	Shanxi Province, China	ITS, SSU	√	Wang et al. (2016)
	SSBG2	*Coccus hesperidum* (Insect)	The South-Siberian Botanical Garden, Russia	ITS	√	Skaptsov et al. (2017)
	TAMA 173	*Aphidoidea* sp. (Insect)	Ibaraki, Japan	ITS		Fukuda et al. (2014)
	CHE-CNRCB 373	*Diaphorina citri* (Insect)	Colima, USA	ITS		Berlanga-Padilla et al. (2018)
	ARSEF 8822	Culicid (Insect)	Tanzania			Hubner-Campos et al. (2013)
	ARSEF7550	Coccoidea (Insect)	Brazil	TEF		GenBank
	1T9BA	Tick (Insect)	New York, USA	ITS		Greengarten et al. (2011)
	Btab03	*Bemisia tabaci* (Insect)	South Korea	ITS		GenBank
	113-8	Mosquitoes (Insect)	Japan	ITS		Ishii et al. (2015)
*S. lanosoniveum*	7S	*Heterodera schachtii* (Nematode)	Iran	ITS		GenBank
		Hair of giant panda (Animal)	China	ITS		GenBank
	2502	Bronchoalveolar lavage fluid (Human)	China	ITS		GenBank
	41559-3	Cave and mine	New York State, USA	ITS, LSU		GenBank
	CBS 321.72		Malaysia	SSU, LSU, ITS		Genbank; Summerbell et al. (2011)
	CBS 322.72		Malaysia	ITS		GenBank

Note: ‘√’ means related data are available. The strains collected from Thailand are indicated with **black bold**.

###### Material examined.

THAILAND, Chiang Mai Province, Mushroom Research Centre, on *Ophiocordyceps unilateralis*, 19 February 2018, *Deping Wei*, MRC18021901 (HKAS 102447; living culture: MFLUCC 18–1385). Sequences generated from this strain have been deposited in GenBank with accession numbers: SSU = MK752791, LSU = MK752849, ITS = MK752683, TEF = MK926450, RPB1 = MK882622.

###### Note.

Our isolate MFLUCC 18–1385 colonised on a decayed *Ophiocordyceps unilateralis* with white hyphae. In a thorough examination of the *Ophiocordyceps unilateralis* host, we found the phialides and conidia of our isolate grown on the surface of the host (Figure 5). Phylogenetically, our isolate grouped with the strains of *Simplicillium lanosoniveum* with high bootstrap support (85% ML, 0.99 BYPP, 67% MP, Figure 2). The nucleotides comparison between our isolate and the type strain of *Simplicillium lanosoniveum* (CBS123.42) showed only 5 bp differences out of 539 in the ITS region. This evidence proves that our isolate is a strain of *S. lanosoniveum*, according to Jeewon and Hyde (2016). Morphologically, it resembles *S. lanosoniveum* with solitary phialides without verticillate branches and conidia adhering on a slimy head. Most of the previous descriptions of this species were given in hand-drawings and scanning electron microscopy (SEM) patterns (Zare and Gams 2001; Ward et al. 2012; Gauthier et al. 2014). *Simplicillium lanosoniveum* has been reported from *Enhalus acoroides* (seagrass) in Trang Province, Thailand. In this study, we introduce our isolate MFLUCC 18–1385 as a new host record of *Simplicillium lanosoniveum* from *Ophiocordyceps unilateralis* and provide the updated morphological features for a better understanding of this species. *Simplicillium lanosoniveum* has been frequently reported as a hyperparasite of rust and plant pathogenic fungi. Therefore, this species has a high potential of being a natural source of microbial agents against microbiological diseases in commercial agriculture (Baiswar et al. 2014; Berlanga-Padilla et al. 2018). At first, we included all available sequences of *S. lanosoniveum* from GenBank in the individual gene tree. Some strains did not group with other strains but distributed throughout the genus in primary analyses (data not shown), so we excluded those strains from the final phylogenetic analysis. Most of the reported strains of *S. lanosoniveum*, including the invalid strains, are listed in Table 2 to show their distribution and host range, as well as the sequence data availability.

**Figure 3.**
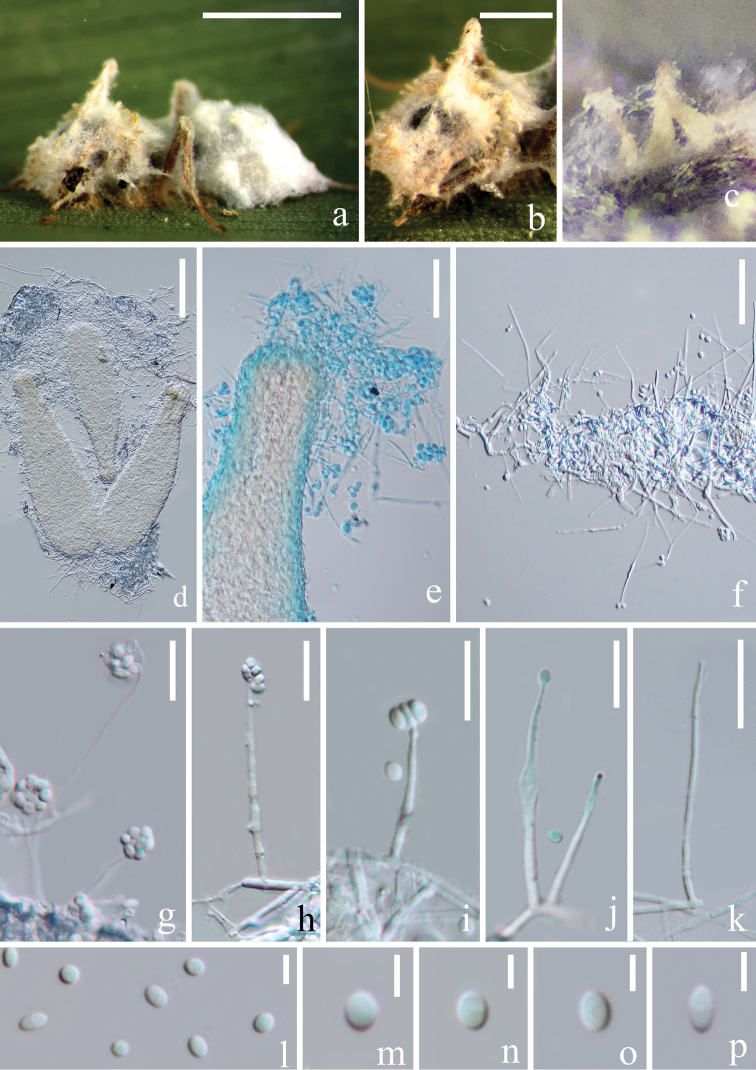
*Simplicillium formicae* (from HKAS 102459, holotype) **a** superficial hyphae associated with the ant host **b–e** flask-shaped synnemata **f–k** phialides bearing conidia **l–p** conidia. Scale bars: 1000 µm (**a**); 500 µm (**b**); 100 µm (**d**); 30 µm (**e, f**); 15 µm (**j, k**); 10 µm (**l–p**) (**e** stained with cotton blue solution).

**Figure 4.**
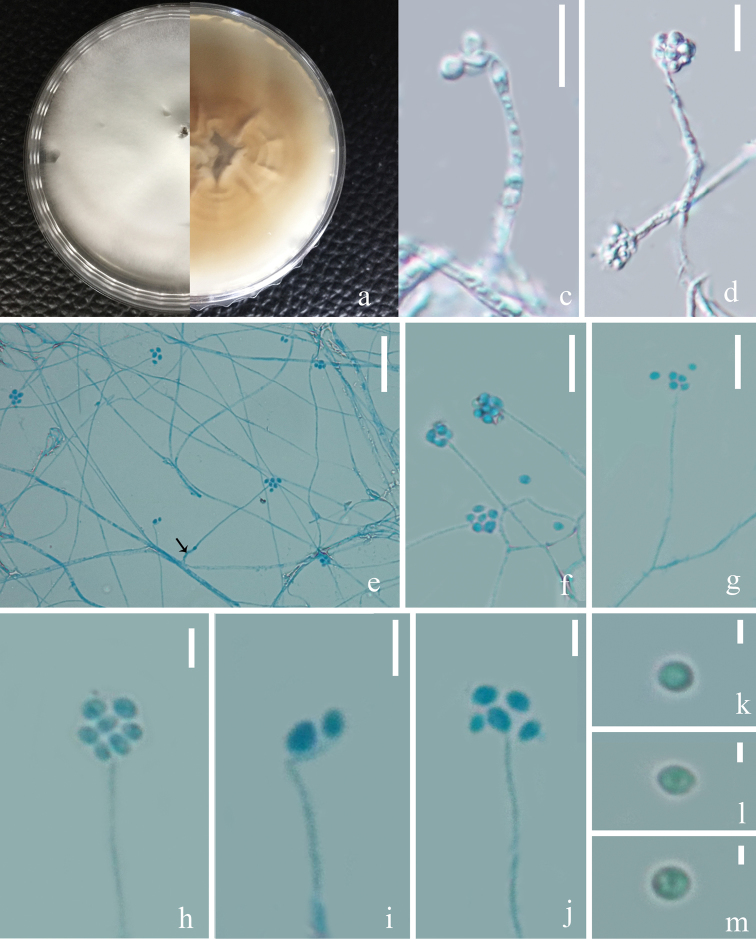
*Simplicillium formicae* (MFLUCC 18–1379, ex-type living culture) **a** upper and reverse view of cultures on PDA after 30 days **e–g** phialides indicated with black arrow **c, d, h–j** conidial mass on the tip of phialides **k–m** conidia. Scale bars: 10 µm (**c, d, f, g**); 20 µm (**e**); 3 µm (**h–j**); 1 µm (**k–m**) (**e–j** stained with cotton blue solution).

**Figure 5.**
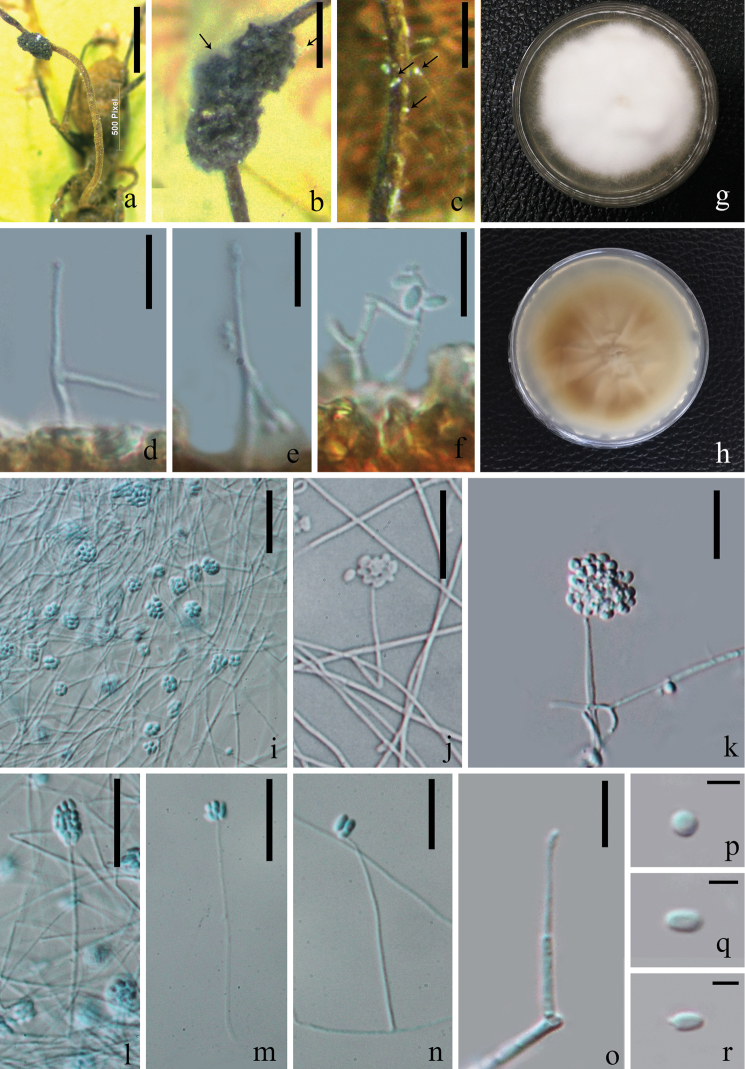
*Simplicillium lanosoniveum***(a–f** from HKAS 102447, **g–r** from MFLUCC 18–1385) **a** host (*Ophiocordyceps unilateralis*); **b, c** hyphae associated with host indicated with black arrows **g, h** upper and reverse view of cultures on PDA after 40 days incubation **i–l** conidial mass on the tip of phialides **m–o** phialides bearing conidia **p–r** conidia. Scale bars: 15 µm (**i–m**); 10 µm (**d–f, n, o**); 3 µm (**p–r**) (**i, l–n** stained with cotton blue solution).

##### Ophiocordyceps unilateralis

Taxon classificationFungiHypocrealesCordycipitaceae

(Tul. & C. Tul.) Petch, Trans. Br. mycol. Soc. 16(1): 74 (1931)

08A33901-9124-5910-8843-1D5824EDF08F

Index Fungorum number: 281145;

Facesoffungi number: FoF 05815

Figure 6

###### Description.

Parasitic on ants (*Formicidae*). ***Sexual morph***: *Stromata* up to 14 mm in length, 0.5 mm wide in the broadest part, cylindrical, brown, slightly tapering towards the apex, single, piercing through the dorsal neck region of the ant host. *Ascomatal cushion* hemisphere, up to 1.2 mm in diam., laterally attaching to the erect stroma stalk, dark brown, with ostioles protruding from the cushions. *Perithecia* 200–400 × 50–120 (xˉ = 294 × 81, n = 10) µm, sub-immersed, flask-shaped. *Asci* and ascospores were too old to observe their features. ***Asexual morph***: Undetermined.

###### Note.

This collection was already decayed and was colonised by other fungi which we introduced as a new host record of *Simplicillium lanosoniveum* from Thailand. The outline of this specimen was intact, while its asci and ascospores were too old to analyse. We retrieved DNA through direct sequencing from the stromal tissue.

Sequences generated from this specimen have been deposited in GenBank with accession numbers: SSU = MK752759, LSU = MK752812, ITS = MK752874. The herbarium material is deposited at KUN herbarium, Yunnan Province, China. In the multi-gene phylogenetic tree, this collection groups with *Ophiocordyceps unilateralis* (OSC 128574) with a strongly supported bootstrap value (100% ML, 1.00 BYPP, 100% MP, Figure 1). Therefore, we identify this collection as *O. unilateralis*, based on its morphologic features and molecular evidence.

**Figure 6.**
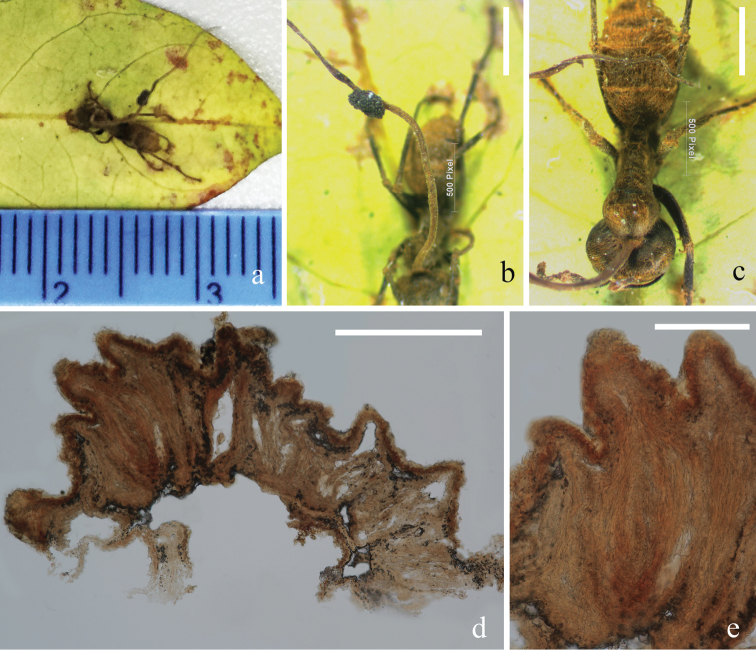
*Ophiocordyceps unilateralis* (from HKAS 102447) **a** stroma emerging from host **b** ascomata on stroma **c** host (Formicidae) **d, e** decayed perithecia. Scale bars: 500 µm (**b, c**); 300 µm (**d**); 100µm (**e**).

#### Key to accepted species of *Simplicillium*

**Table:** 

1a	Conidia formed in sympodia	***S. sympodiophorum***
1b	Conidia solitary, borne on the tip of phialides	***S. calcicole***
1c	Conidia aggregate in chains	**2**
1d	Conidia aggregate in subglobose to ellipsoidal heads	**3**
1e	Conidia aggregate in globose heads	**4**
2a	Conidia 2.5–3.5 × 1–2 µm, obclavate to ellipsoidal, formed in short imbricate chains	***S. obclavatum***
2b	Conidia 3.5–5.0 × 1.0–1.5 μm, oval, ellipsoidal or cylindrical, formed in vertical chains	***S. chinense***
2c	Conidia 7.2–12.5 × 1 µm, long, fusiform to short filiform, hyaline, straight to curved, formed in vertical chains	***S. filiforme***
3a	Phialides 15–50 × 0.7–1.0 µm, colonies light to dark-brown reverse on PDA, usually with yellow pigment diffusing into the agar	***S. lamellicola***
3b	Phialides 11–44 (–70) × 1.0–2.4 µm, colonies cream-coloured reverse on PDA, no diffused pigment	***S. coffeanum***
4a	Present flask-shaped synnemata	***S. formicae***
4b	Synnemata absent	**5**
5a	Conidia cylindrical	**6**
5b	Conidia globose to subglobose or ellipsoidal	**7**
6a	Phialides 23–53 × 1.2–2.0 µm, long	***S. cylindrosporum***
6b	Phialides 17–32 × 1.2–2.0(–2.5) µm, short	***S. aogashima***
7a	Phialides 35–75 × 1.2–3.0 µm, conidia 4.5–6.0 × 2.5–3.5 µm, colonies light yellow to deep tawny in reverse view on PDA	**S. lanosoniveum var. tianjinensis**
7b	Phialides 15–39 × 0.7–1.9 µm, conidia 1.5–3 × 0.7–1.3 µm, colonies brownish-cream to pale yellow reverse on PDA	***S. lanosoniveum***
7c	Phialides 11–31(–47) × 1.0–1.7 µm, conidia 2.0–3.5 × 1.8–2.5(–2.8) µm, colonies brown reverse on PDA	***S. minatense***
7d	Phialides (15–)20–42(–50) × 1.0–2.3 µm; conidia 2.3–4.0(–4.5) × 1.5–3.3 µm, colonies brownish-orange to brown reverse on PDA	***S. subtropicum***

## Conclusion

A new species *Simplicillium formicae* and a new host record species *Simplicillium lanosoniveum* from *Ophiocordyceps unilateralis* were introduced, based on phylogenetic analyses and morphological evidence. The host and distribution of *S. lanosoniveum* was summarised and a key to *Simplicillium* was provided.

## Supplementary Material

XML Treatment for Simplicillium formicae

XML Treatment for Simplicillium lanosoniveum

XML Treatment for Ophiocordyceps unilateralis
